# Chemical and sensory characterization of the apple-like aroma in ‘Fenza 1’ banana unveiled by sensory-directed analysis and molecular modeling

**DOI:** 10.1016/j.fochx.2026.104047

**Published:** 2026-05-28

**Authors:** Wen Zeng, Zhiwen Zeng, Tao Wang, Yacong Hou, Youfeng Jiang, Dafeng Dong, Tianxiang Li, Lufeng Fu, Ying Liu, Xuesong Cao, Zhuo Chen, Peitao Lü

**Affiliations:** a State Key Laboratory of Tropical Crop Breeding, Institute of Tropical Bioscience and Biotechnology & Sanya Research Institute, Chinese Academy of Tropical Agricultural Sciences, Sanya 572024, Hainan, China; b College of Horticulture, Horticultural Plant Biology and Metabolomics Center, Haixia Institute of Science and Technology, Fujian Agriculture and Forestry University, Fuzhou 350002, Fujian, China

**Keywords:** Banana aroma, HS-SPME-GC–MS, GC-O, Molecular docking, Molecular dynamics, Aroma addition test

## Abstract

The unique apple-like aroma of the ‘Fenza 1’ (FZ1) banana has long been valued by consumers, yet the underlying chemical basis remains poorly understood. Therefore, this study aimed to investigate the specific compounds and mechanisms behind this distinctive aroma phenotype using a multi-faceted approach. Descriptive sensory analysis revealed that FZ1 displayed an apple-like aroma intensity intermediate between the common ‘Baxi’ (BX) banana and the ‘Ruixue’ (RX) apple. Headspace solid-phase microextraction gas chromatography–mass spectrometry (HS-SPME-GC–MS) and gas chromatography-olfactometry (GC-O) analyses identified 17 aroma-active compounds contributing to the aroma variations among the three fruits. Among these, hexyl acetate (OAV = 58), trans-2-hexenal (OAV = 502), and hexanal (OAV = 1191) were identified as key contributors to the apple-like aroma. Aroma addition experiments further demonstrated that these compounds, particularly in combination, enhanced the perception of apple-like aroma within a banana matrix. Molecular docking and molecular dynamics simulations suggested stable interactions between these compounds and specific human olfactory receptors via hydrophobic interactions and hydrogen bonding. These findings clarify the chemical basis of this distinctive aroma and can guide flavor enhancement strategies for bananas.

## Highlights

• The apple-like aroma of ‘Fenza 1’ banana was characterized via sensory and chemical analysis.
• Hexyl acetate, 2-hexenal, and hexanal were identified as key aroma-active compounds.
• Molecular docking revealed stable binding to olfactory receptors via hydrophobic/hydrogen-bond interactions.
• Molecular dynamics simulations confirmed the stability of ligand-receptor complexes.
• Aroma recombination tests validated the synergistic effect of the three key compounds.

## 1 Introduction

Banana (*Musa* spp.), recognized as the world's second most important fruit crop, serves as both a vital food staple and a significant economic commodity in tropical and subtropical regions (Dale et al., 2017). China ranks among the top three global producers of banana, whose production substantially influences the international banana industry (Zuo et al., 2017). Among the prominent cultivars in China are the ‘Baxi’ banana (*Musa* spp. AAA group) and the ‘Fenjiao’ banana (*Musa* spp. ABB group) (Dou et al., 2020). The latter is particularly valued by consumers for its distinct apple-like aroma.

Aroma is a critical determinant of fruit flavor quality (Song et al., 1997; Li et al., 2023). Extensive research has established that not all volatile compounds contribute equally to perceived aroma; only a select group of key odorants are capable of binding to olfactory receptors and subsequently participating in the formation of specific aroma perceptions in the brain (Pino & Febles, 2013; Chen et al., 2021). While nearly 250 volatile compounds have been identified in fresh bananas and processed banana products (Pino & Febles, 2013), only a fraction of these contribute to the characteristic banana aroma.

The volatile profile of bananas is predominantly composed of esters, alcohols, and ketones. Esters, particularly acetate esters such as isoamyl acetate and isobutyl acetate, as well as various butanoate esters, are recognized as major contributors to the typical aroma of ripe bananas (EI Hadi et al., 2013; Marriott, 1980). The aroma composition of banana fruit is influenced by a multitude of factors, including genotype (Aurore et al., 2011; Bugaud & Alter, 2016), cultivation conditions (Dong et al., 2015), ripening stage (Zhu et al., 2018), harvest season (Shivashankara et al., 2017), and postharvest storage practices (Facundo et al., 2012; Zhu et al., 2020). These factors modulate the composition and abundance of volatile compounds, thereby shaping the final aroma characteristics and sensory quality of the fruit. However, the vast majority of existing studies have predominantly focused on the typical ‘banana-like’ aroma profiles of standard Cavendish-type cultivars. Consequently, the chemical and molecular basis of atypical, high-value flavor phenotypes—such as the distinctive apple-like aroma of the ‘Fenjiao’ banana—remains critically understudied. This specific research gap not only limits our fundamental understanding of chemodiversity within *Musa* species but also hinders targeted breeding efforts aimed at flavor enhancement.

To address this gap, this study provides a novel, comprehensive investigation to identify the key aroma-active compounds underpinning the distinctive apple-like aroma of the ‘Fenza 1’ (FZ1) banana, a cultivar of ‘Fenjiao’ (Yang et al., 2022). Initially, an untargeted analysis of volatile compounds was performed using HS-SPME-GC–MS. Subsequently, the combination of odor activity value (OAV) calculation and gas chromatography-olfactometry (GC-O) was then employed to pinpoint aroma-active compounds with significant olfactory impact, and aroma flavor wheels were constructed for each sample type. Key aroma-active compounds were subsequently identified based on multivariate analysis integrating the chemical and sensory data. The contribution of these compounds to apple-like aroma perception in a banana matrix was further evaluated through aroma addition experiments. Finally, molecular docking and molecular dynamics simulations were used to explore potential interactions between the characteristic aroma compounds and human olfactory receptors. To our knowledge, this is the first study to integrate multidimensional analytical chemistry, sensory evaluation, and molecular dynamics to elucidate the human receptor-level interactions of banana aroma compounds. Collectively, these findings provide a theoretical foundation for the targeted improvement of banana flavor and the development of specific banana flavorings.

## 2 Materials and methods

### 2.1 Materials and chemicals

Plant Materials: Banana fruits of two cultivars, ‘Baxi’ (*Musa* spp. AAA group, banana-like) and ‘Fenza 1’ (*Musa* spp. ABBB, Pisang Awak subgroup, apple like), were harvested from a plantation in Danzhou, Hainan Province, China. One apple cultivar, ‘Ruixue’ was obtained separately. All banana bunches were collected at the same physiological maturity stage. After harvest, the banana was air-dried and then immersed in a 0.1% (v/v) Sporgon solution for 5 min. Subsequently, the fruits were removed and air-dried again at room temperature. To initiate ripening, the pre-treated fruits were immersed in a 0.1% (w/v) ethephon solution for 5 min (Li et al., 2025; Zhu et al., 2023). At five different time points post-treatment (0, 1, 3, 5, and 7 days), peel color and pulp firmness were measured (eight independent fruits were randomly selected per group as biological replicates). Overall sensory acceptability (comprehensive evaluation of sweetness, sourness, aroma, and firmness) was assessed according to Bugaud et al. (2016). All samples were peeled, sliced, immediately frozen in liquid nitrogen, and stored at −80 °C for further analysis.

Chemicals: A standard n-alkane mixture (C7–C40) was purchased from O2SI (Shanghai, China). 2-Octanol (chromatographic grade; purity ≥99.8%) was obtained from Sigma-Aldrich (Shanghai, China). *trans*-2-Hexenal (purity = 98%), hexyl acetate (purity = 98%), and hexanal (purity = 98%) were obtained from Aladdin (Shanghai, China).

### 2.2 Descriptive sensory analysis

#### 2.2.1 Panel training

The sensory evaluation panel consisted of 10 trained assessors (5 males and 5 females, aged between 22 and 51 years) specializing in aroma evaluation. The panel received training twice a week for four weeks. Each session focused on two aspects: (1) recognition and memorization of the six target aroma notes using reference standards, and (2) calibration of the 6-point intensity scale. Reference standards were prepared by dissolving each compound in a suitable solvent and presenting them on smelling strips at a concentration that gave a clear, moderate intensity. The specific reference compounds used were: “fruity” (ethyl butyrate), “sweet” (vanillin), “grassy” (cis-3-hexen-1-ol), “acidic” (citric acid), “banana-like” (isoamyl acetate), and “apple-like” (hexyl acetate). Panelists were asked to sniff each reference and associate it with its descriptor. In subsequent sessions, they practiced scoring real fruit samples using the scale until their individual results were repeatable and the panel's overall performance was consistent (*p* ≥ 0.05). The training was considered complete when panelists could accurately, skillfully, and consistently use the descriptive terms and sensory scales.

Ethical Statement: Ethical approval was not required for this sensory evaluation study, as it involved standard food sensory analysis procedures using non-invasive methods and posed no risks to the participants' health, in accordance with the guidelines of Chinese Academy of Tropical Agricultural Sciences. All participants were fully informed of the study procedures, and written informed consent was obtained from each participant before the evaluation. The research was conducted in compliance with the Declaration of Helsinki, and the privacy and rights of all participants were strictly protected, with no personal identification information disclosed.

#### 2.2.2 Development of aroma descriptors

To accurately evaluate the aroma profiles of the samples, the sensory panel, through discussion, ultimately selected six aroma descriptors with a frequency of use >80% to characterize the overall aroma: “fruity” (ethyl butyrate-like, generic ripe fruit), “sweet” (vanillin-like, vanilla sweet), “grassy” (cis-3-hexen-1-ol-like, fresh-cut grass), “acidic” (citric acid, lemon), “banana-like” (isoamyl acetate-like, ripe banana), and “apple-like” (hexyl acetate-like, crisp apple). The panel was thoroughly familiarized with these definitions and references during the training phase (Section 2.2.1).

#### 2.2.3 Scaling of sensory intensity

The intensity of sample aromas was assessed using a 6-point scale, consistent with the GC-O method: 0 = not detectable, 1 = weak, 2 = moderate, 3 = medium, 4 = strong, 5 = intense. Each assessor evaluated the samples independently without discussion, and three replicates were performed. The average score was taken as the final result. The ethical approval procedure was the same as for the sensory evaluation.

### 2.3 Untargeted HS-SPME-GC–MS analysis of sample volatile compounds

The untargeted GC–MS analysis of VOC in the samples was conducted following the well-established protocol from a previous study (Li et al., 2025; Zhu et al., 2023) with minor modification. A 65 μm polydimethylsiloxane/divinylbenzene (PDMS/DVB) fiber (Supelco, Bellefonte, PA, USA) was used. Briefly, 1.0 g of freeze-dried banana powder was weighed into a 20 mL headspace vial, and 4 mL of distilled water was added. The sample was incubated at 60 °C for 15 min, extracted for 30 min, and desorbed for 5 min.

Analysis of VOCs was performed using a TRACE 1300 ISQ DQ gas chromatography–mass spectrometry system (Thermo Fisher Scientific, USA). Chromatographic separation was achieved using an Agilent DB-5MS capillary column (30 m × 0.25 mm, 0.25 μm). High-purity helium (≥ 99.999%) was used as the carrier gas at a constant linear velocity of 36.80 cm/s. The injector temperature was maintained at 250 °C, and the injection was performed in splitless mode.

The GC temperature program was as follows: initial temperature 40 °C held for 1 min; increased to 60 °C at 2 °C/min, held for 2 min; then raised to 180 °C at 10 °C/min, held for 5 min; finally increased to 220 °C at 5 °C/min, held for 5 min. MS conditions: electron impact (EI) ionization source at 70 eV; ion source temperature 230 °C; transfer line temperature 250 °C. Data acquisition was performed in full scan mode with a mass range of *m*/*z* 45–500 and a solvent delay of 3 min.

To ensure highly accurate identification, volatile compounds were confirmed using a rigorous triple-validation approach: matching mass spectra with the NIST standard library, comparing experimentally calculated retention indices (RIs) with reference literature values, and cross-referencing odor descriptions obtained via gas chromatography-olfactometry (GC-O). For quantitative analysis, a semi-quantitative approach utilizing 2-octanol as an internal standard was employed. This method was selected as it is a widely established and robust approach for evaluating the relative abundance and comparative variations of volatile profiles across different fruit cultivars.

### 2.4 Analysis of odor-active compounds by gas chromatography-olfactometry (GC-O)

Odor characteristics of volatile organic compounds were analyzed using a gas chromatography-olfactometry system, which consisted of the aforementioned GC–MS instrument (TRACE 1300 ISQ, Thermo Fisher Scientific) coupled with a Shimadzu ODE-2030 olfactory detection port (Shimadzu, Japan). Sample preparation procedures and chromatographic parameters (including injection mode, carrier gas flow rate, and temperature program) were consistent with those described in Section 2.3 for GC–MS analysis to ensure comparability of results.

Based on pre-training results, five assessors with high olfactory acuity were selected for GC-O evaluation. During the sniffing process, the assessors recorded the sensory descriptors and corresponding intensity scores for each odor-active peak. The average odor intensity value was calculated for subsequent analysis.

### 2.5 Molecular docking and molecular dynamic simulation analysis

Molecular docking was employed to investigate the interaction mechanisms between three key volatile compounds in ‘Fenza 1’ banana – hexanal, hexyl acetate, and trans-2-hexenal – and olfactory receptors. The 3D structure files (SDF format) of the target compounds were obtained from the PubChem database (https://pubchem.ncbi.nlm.nih.gov/). The 3D structural models of the olfactory receptors were sourced from the UniProt database (Table S1). Molecular docking simulations were performed using AutoDock Vina 1.2.5, with the exhaustiveness parameter set to 8. Docking results were primarily screened for valid conformations based on binding energy (kcal/mol) and conformational stability (root mean square deviation, RMSD < 2 Å). Finally, the interaction patterns were visualized using Discovery Studio software.

To further investigate the dynamic binding characteristics of ligand-receptor complexes, molecular dynamics (MD) simulations were performed. Six complex systems (Hexanal-O60404, Hexanal-P59827, trans-2-hexenal-P59827, trans-2-hexenal-Q8NGA5, Hexyl-acetate-Q8NGF6, and Hexyl-acetate-Q8NGX5) were selected for study. Simulations were conducted using the GROMACS 2020.6 software platform, with the AMBER99SB force field and the SPC water model. The time step was set to 2 fs, and long-range electrostatic interactions were treated using the Particle Mesh Ewald (PME) method. The systems were first equilibrated for 2 ns in the NVT ensemble, followed by 2 ns of equilibration in the NPT ensemble at a constant temperature of 300 K. Finally, a 100 ns production MD simulation was performed, with a non-bonded interaction cutoff set to 1.0 nm.

### 2.6 Aroma addition experiment

#### 2.6.1 Matrix for addition experiments

Freeze-dried powder prepared from the same batch of ‘Baxi’ bananas as the test samples was used to maximize the retention of original fruit components and volatile compounds, while minimizing batch-to-batch variation. The freeze-dried powder was rehydrated to form a homogenate, which served as the matrix for the aroma addition experiments. Rehydration was performed using 4 °C redistilled water (odor-free) as the solvent, with low temperature used to reduce potential enzymatic reactions or chemical changes. A precise amount of freeze-dried powder was weighed into a sealed headspace vial, and redistilled water was added in a ratio corresponding to the dry matter content of the fresh fruit (e.g., for ‘Baxi’ banana with 22.6% dry matter content, 22.6 g of freeze-dried powder was mixed with 77.4 g of water). After adding water, the vial was immediately sealed, briefly vortex-mixed at high speed, and then allowed to stand for 15–20 min for complete hydration, resulting in a homogeneous banana homogenate. Homogenate without added target aroma compounds served as the negative control, and pure water served as the blank control.

#### 2.6.2 Setting the concentration of added compounds

Referring to previous methods (Wei et al., 2025), the target aroma compounds were added to the homogenate at concentrations listed in Table S2. The concentrations of the three compounds (hexanal, hexyl acetate, and trans-2-hexenal) found in the ‘Fenza 1’ banana samples were designated as the medium dose (M). Low (L) and high (H) doses were set at 50% and 200% of the M concentration, respectively. After mixing, the samples were subjected to triangle tests by a trained panel to confirm significant differences (*p* < 0.05) between the spiked samples and the control sample. Subsequently, the apple-like aroma intensity was scored using the 5-point scale. All sensory experiments were performed by the same trained panel.

### 2.7 Statistical analysis

All statistical analyses were performed using SPSS Statistics 26.0 software (IBM, USA). Data are presented as mean ± standard deviation (mean ± SD). Differences between groups were compared using one-way analysis of variance (ANOVA). If homogeneity of variance was confirmed, the Least Significant Difference (LSD) post-hoc test was used; otherwise, Dunnett's T3 test was applied. The statistical significance level was set at *p* < 0.05. Metabolomics data were analyzed using the MetaboAnalyst 5.0 online platform (https://www.metaboanalyst.ca/). Bar graphs and line charts were generated using GraphPad Prism 8.0 software, while radar charts and aroma feature profiles were created using Origin 2021 software.

## 3 Results and discussion

### 3.1 Sensory characteristics of BX, FZ1 and RX

**Fig. 1:**
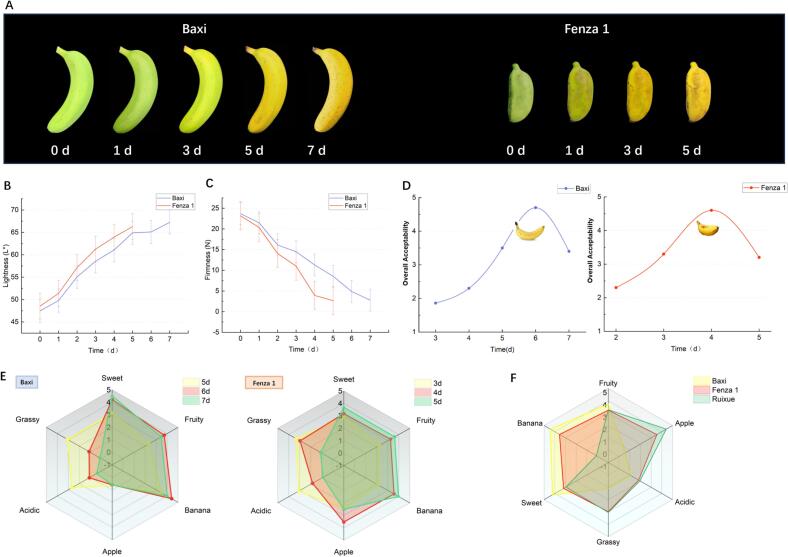
Changes in physicochemical properties and sensory profiles of Baxi (BX) and Fenza 1 (FZ1) bananas during ripening. (A) Visual appearance. (B) Peel lightness (L*). (C) Fruit firmness. (D) Overall acceptability scores. (E) Descriptive sensory analysis radar charts at different ripening stages. (F) Descriptive sensory analysis of BX, FZ1 and RX.

To identify a representative ripening stage for subsequent analyses, BX (‘Baxi’, banana-like) and FZ1 (‘Fenza 1’, apple-like) bananas were monitored for physiological and sensory changes during ripening. We measured the color difference and firmness of two banana cultivars, BX and FZ1 (Fig. 1A), during ripening and evaluated their overall sensory acceptability. The results showed that the peel color lightness (L* value) of both cultivars gradually increased (Fig. 1B), while the firmness of the fruits gradually decreased during ripening (Fig. 1C). The rate of change was greater for FZ1 than for BX; the firmness of FZ1 dropped below 3 N by day 5, whereas BX reached this level only on day 7, indicating a shorter ripening period for FZ1.

Overall sensory acceptability was further evaluated to determine the optimal stage for consumption. Acceptability scores exceeded 3 (on a 6-point scale) after day 5 for BX and after day 3 for FZ1, indicating those stages met the criteria for acceptable eating quality (Fig. 1E). The highest acceptability was observed on day 6 for BX and day 4 for FZ1. Sensory attribute evaluation also confirmed that BX displayed the strongest banana-like and fruity aromas on day 6, whereas FZ1 exhibited the most pronounced apple-like aroma on day 4 (Fig. 1E). These time points were therefore selected for subsequent descriptive sensory analysis.

A trained panel conducted descriptive sensory analysis following principles of quantitative descriptive analysis, with a modified intensity scale. The data revealed clear differences in the aroma profiles aroma among BX, FZ1, and RX, (Fig. 1F). FZ1 exhibited a distinct apple-like aroma that was readily distinguishable from BX but less intense than RX. In addition to the apple-like note, FZ1 showed pronounced green and sweet aromas, with intensities comparable to those observed in RX, giving FZ1 an overall aroma profile resembling that of green apple. In contrast, BX presented a relatively simpler aroma profile dominated was characterized mainly by banana-like, sweet, and fruity aromas (Fig. 1F). The three samples exhibited clear differences in the intensity of the apple-like aroma (RX > FZ1 > BX).

### 3.2 Untargeted GC–MS of BX, FZ1 and RX

**Fig. 2:**
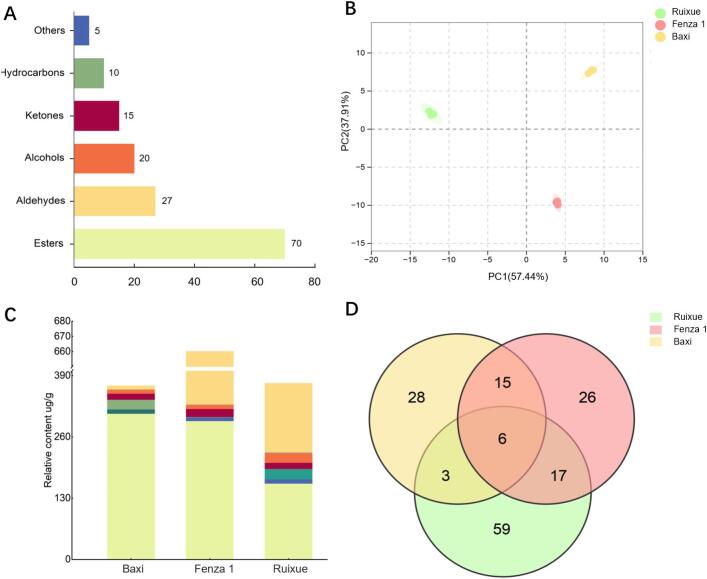
Comparative analysis of aroma characteristics and volatile compounds in BX, FZ1, and RX. (A) Number distribution of volatile compounds. (B) Principal component analysis (PCA) score plot. (C) Concentration distribution of different volatile classes. (D) Venn diagram of volatile compounds detected in each sample group.

Untargeted analysis was performed on the three sample groups: apple-like banana “FZ1”, banana-like banana “BX” and apple “RX” using HS-SPME-GC–MS. In total, 154 volatile compounds were identified by comparing their mass spectra and retention indices with those in the NIST 2017 database, a house database, and standard reference materials (Table S3, Fig. S1), primarily including esters (70 compounds), aldehydes (27 compounds), alcohols (20 compounds), ketones (13 compounds), and hydrocarbons (10 compounds) (Fig. 2A).

Principal Component Analysis (PCA) revealed that the first two principal components accounted for 95.35% of the total variance (PC1 = 57.44%, PC2 = 37.91%), effectively differentiating the three sample types (Fig. 2B). FZ1 has a much higher total volatile intensity compared to BX and RX (Fig. 2C). Ven diagram analysis indicated that the three samples shared 6 common aroma compounds. Specifically, BX and FZ1 shared 15 compounds, FZ1 and RX shared 17 compounds, while BX and RX shared only 3 compounds. BX contained 28 unique compounds, FZ1 contained 26, and RX contained 59 unique compounds (Fig. 2D). Given that both BX and FZ1 are banana fruits, their volatile compound compositions showed relatively high similarity. The overlap of compounds between RX and FZ1 was higher than that between RX and BX, a result consistent with the descriptive sensory analysis finding that the aroma profiles of FZ1 and BX were relatively similar. Esters are primary contributors to banana-like and fruity aromas (Liao et al., 2020), while aldehydes play a key role in forming apple-like and green aromas (Yan et al., 2020). Consistent with this, the sensory analysis found that the aroma profile of BX was dominated by banana-like and fruity notes, whereas both FZ1 and RX exhibited distinct green and apple-like aroma characteristics, which aligns well with the distribution pattern of volatile compounds described above.

### 3.3 Analysis of aroma compounds in BX, FZ1, and RX

**Fig. 3:**
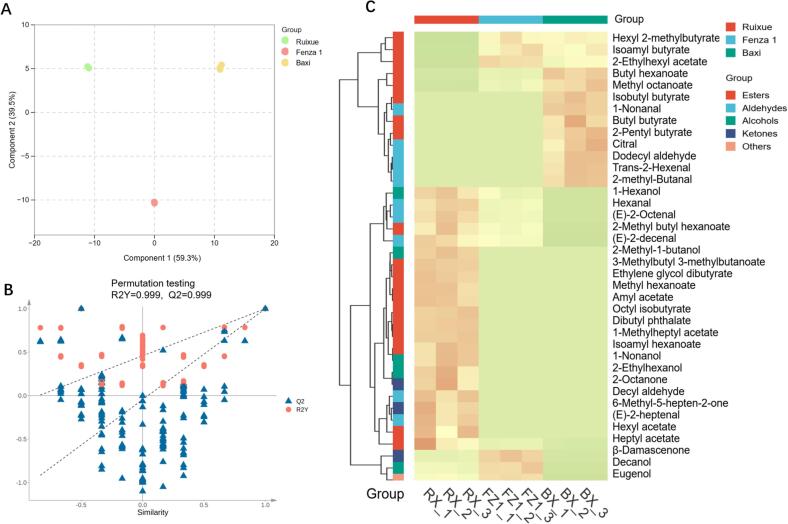
Multivariate analysis of volatile compounds in BX, FZ1, and RX. (A) Orthogonal partial least squares-discriminant analysis (OPLS-DA) score plot. (B) Permutation test plot for the OPLS-DA model validation. (C) Heatmap of key aroma-active compounds (VIP > 1 and OAV > 1).

To elucidate the aroma differences among BX, FZ1, and RX, we performed a systematic analysis of their volatile compound compositions and identified differential compounds responsible for their distinct aroma profiles. Orthogonal Partial Least Squares-Discriminant Analysis (OPLS-DA) was employed to analyze the differences in volatile compounds among the three sample types. The OPLS-DA revealed clear separations among the three samples (Component 1 = 59.3%, Component 2 = 39.5%), suggesting significant disparities in their volatile compositions (Fig. 3A). The model demonstrated high reliability (R^2^Y = 0.997, Q^2^ = 0.990), and the Q^2^ regression line intersected the zero point below the vertical axis, indicating no overfitting in the model (Fig. 3B).The Variable Importance in Projection (VIP) value was used to evaluate the contribution of each component to the classification and discrimination. A VIP > 1.0 was considered a significant indicator for sample differentiation (Wang et al., 2022). Based on the criterion of VIP > 1.0, a total of 116 differential compounds were screened (Table S4).

Considering not all 116 compounds will have equal contribution to the odor of the fruit, OAVs were calculated based on semi-quantitative concentrations estimated in the GC–MS analysis using the internal standard, assuming a response factor of 1. Since the nature of this study is to profile fruit aroma and standards are unavailable for most of the compounds discovered, this approach is generally considered acceptable and widely used in other similar aroma characteristic studies (Coleman et al., 2022; Nuzzi et al., 2008). Compounds with OAV > 1 are considered to significantly contribute to the aroma profile (Liao et al., 2020). This study identified 38 aroma compounds that simultaneously satisfied OAV > 1 and VIP > 1 (Fig. 3C; Table 1), including 19 esters and 10 aldehydes. It is particularly noteworthy that hexanal (OAV = 791 in RX, OAV = 1191 in FZ1), hexyl acetate (OAV = 791 in RX, OAV = 1191 in FZ1), trans-2-hexenal (OAV = 137 in RX, OAV = 502 in FZ1), and nonanal (OAV = 54 in RX, OAV = 25 in FZ1) exhibited high OAV values in both RX and FZ1. These compounds have been confirmed by previous studies as key contributors to apple aroma (De Souza et al., 2016; Zheng et al., 2025).

**Table 1:** Volatile aroma compounds with VIP >1 and OAV > 1.

No.	#CAS	Volatile compounds	Content (ug/g)		
			BX	FZ1	RX
1	23726-93-4	*β*-Damascenone	ND	ND	0.27 ± 0.06a
2	97-53-0	Eugenol	0.22 ± 0.04a	ND	ND
3	2548-87-0	(E)-2-Octenal	ND	ND	1.24 ± 0.11a
4	96-17-3	2-methyl-Butanal	8.61 ± 1.28a	ND	ND
5	3913-81-3	(E)-2-decenal	ND	ND	1.23 ± 0.04a
6	109-15-9	Octyl isobutyrate	ND	ND	0.06 ± 0.01a
7	143-08-8	1-Nonanol	ND	0.20 ± 0.06b	0.42 ± 0.06a
8	10032-15-2	Hexyl 2-methylbutyrate	ND	ND	1.95 ± 0.18a
9	2601-13-0	2-Methylbutyl hexanoate	ND	ND	1.02 ± 0.18a
10	112-54-9	Dodecyl aldehyde	ND	ND	0.54 ± 0.14a
11	5392-40-5	Citral	ND	ND	0.32 ± 0.10a
12	103-09-3	Ethylhexyl esteracetate	0.81 ± 0.25a	ND	ND
13	110-93-0	Methylheptenone	0.79 ± 0.21b	0.79 ± 0.09b	9.71 ± 0.55a
14	18829-55-5	(E)-2-heptenal	ND	ND	1.39 ± 0.18a
15	111-13-7	2-Octanone	9.85 ± 0.56a	ND	ND
16	539-90-2	Isobutyl butyrate	1.15 ± 0.33a	ND	ND
17	106-70-7	Methyl hexanoate	ND	ND	0.25 ± 0.09a
18	112-30-1	Decanol	ND	ND	0.16 ± 0.05a
19	66-25-1	Hexanal	ND	86.93 ± 2.38a	57.78 ± 3.42b
20	628-63-7	Amyl acetate	ND	ND	0.66 ± 0.03a
21	2051-50-5	1-Methylheptyl acetate	1.57 ± 0.32a	ND	ND
22	142-92-7	Hexyl acetate	ND	6.56 ± 0.60b	13.05 ± 1.33a
23	105-72-6	Ethylene glycol dibutyrate	1.01 ± 0.22a	ND	ND
24	111-11-5	Methyl octanoate	ND	ND	0.14 ± 0.02a
25	124-19-6	1-Nonanal	3.62 ± 0.33b	6.54 ± 0.26b	14.11 ± 4.80a
26	84-74-2	Dibutyl phthalate	ND	ND	2.87 ± 0.08a
27	111-27-3	1-Hexanol	ND	3.67 ± 0.29b	7.87 ± 0.87a
28	112-06-1	Heptyl acetat	1.46 ± 0.15a	ND	ND
29	6728-26-3	2-Hexenal	ND	215.01 ± 16.45a	58.71 ± 4.42b
30	626-82-4	Butyl hexanoate	ND	2.05 ± 0.18a	1.00 ± 0.13b
31	137-32-6	2-Methyl-1-butanol	ND	ND	4.22 ± 0.62a
32	104-76-7	2-Ethylhexanol	ND	ND	1.24 ± 0.24a
33	106-27-4	Isoamyl butyrate	136.31 ± 9.86a	39.52 ± 2.34b	ND
34	109-21-7	Butyl butyrate	16.43 ± 1.96a	7.91 ± 0.85b	1.22 ± 0.16c
35	112-31-2	Decyl aldehyde	ND	2.64 ± 0.27b	4.00 ± 0.68a
36	2198-61-0	Isoamyl hexanoate	4.41 ± 0.19a	4.69 ± 0.68a	ND
37	60415-61-4	2-Pentyl Butyrate	11.29 ± 1.38a	12.15 ± 2.91a	ND
38	659-70-1	Isoamyl valerianate	5.40 ± 0.82b	8.61 ± 0.38a	ND

Values are expressed as mean ± standard deviation (*n* = 3). Different lowercase letters (a, b, c) within the same row indicate significant differences between samples at p < 0.05, as determined by one-way ANOVA followed by the Least Significant Difference (LSD) or Dunnett's T3 post-hoc test.

### 3.4 GC-O analysis of aroma components in BX, FZ1, and RX

To discriminate between abundant volatiles and those with true sensory relevance, GC-O was employed to identify odor-active compounds that contribute directly to human perception, as a complement to GC–MS-based volatile profiling and OAC analysis. We conducted GC-O analysis on BX, FZ and RX. In total, 29 odor-active compounds were detected, with FZ1 containing the greatest diversity (23 compounds), followed by RX (16) and BX (14) (Table S5). These compounds collectively constituted the overall aroma profile, including fruity, banana-like, apple-like, green, and sweet notes, showing high consistency with the descriptive sensory analysis results.

The odor-active compounds found in “BX” (banana-like banana), include isoamyl butyrate (fruity), butyl butyrate (fruity, banana), and isoamyl acetate (sweet, banana), which algins with its rich fruity and banana-like base notes. In FZ1 (apple-like banana), the compounds types were more diverse, including isoamyl acetate (banana, sweet), trans-2-hexenal (green, grassy), hexanal (fresh, green, grassy), hexyl acetate (fruity, apple, sweet) and 2-pentanol, acetate (fruity, banana). In RX (apple) was dominated by aldehydes and esters, including hexyl acetate (fruity, apple, sweet), hexanal (fresh, green, grassy), trans-2-hexenal (green, grassy), 1-nonanal (rose, fresh), and methylheptenone (citrus, green). These compounds collectively constituted the overall aroma profile, including fruity, banana-like, apple-like, green, and sweet notes, showing high consistency with the descriptive sensory analysis results.

Significantly, hexyl acetate, *trans*-2-hexenal, and hexanal demonstrated high sensory intensities in both FZ1 and RX. Their status as key differentiating compounds (VIP > 1) with high odor activity (OAV > 1) further validates their fundamental role in defining the aroma profile across both quantitative and sensory dimensions. Among them, hexyl acetate is considered one of the most important contributors to the characteristic apple aroma (Young et al., 1996). Furthermore, as a high character impact compound, it can significantly enhance the perception of apple-like aroma in aroma mixtures (Bult et al., 2002). The GC-O results in this study directly confirmed that hexyl acetate presented strong fruity and apple-like aromas in FZ1 and RX, strongly supporting its role as a core contributor to the apple-like aroma in these samples. Hexyl acetate was perceived as fruity and apple-like in the FZ1 and RX samples, suggesting its likely contribution to the apple-like aroma of the samples. Additionally, the sensory intensities of trans-2-hexenal and hexanal in FZ1 and RX were higher than in BX, primarily imparting fresh, green, and grassy aroma notes. Hexanal is listed as a key volatile compound in apples, associated with green and grassy notes in apples (Jesenko et al., 2025). *trans*-2-Hexenal is also an important component of the characteristic apple-like aroma, contributing green, grassy, and leafy notes, but is softer than hexanal. Comparing the two, hexanal tends to impart a stronger green note, while trans-2-hexenal provides a more subtle leafy and fruity nuance (Espino-Díaz et al., 2016).

### 3.5 Characteristic metabolites for distinguishing BX, FZ1, and RX

**Fig. 4:**
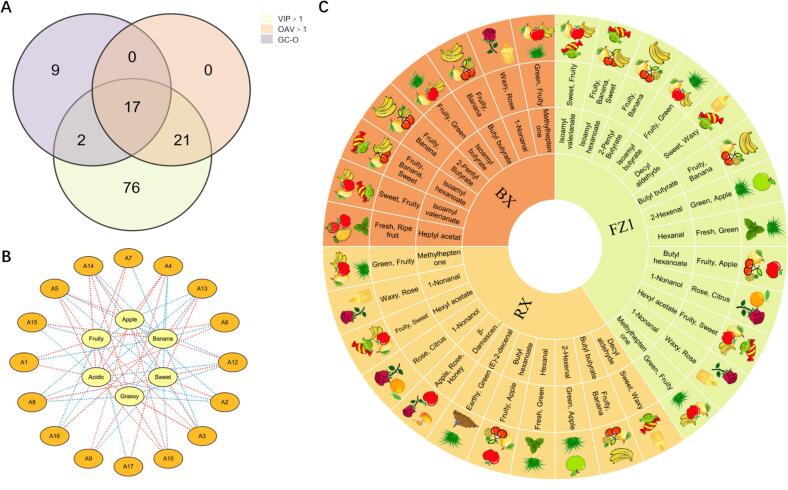
Analysis of aromatic active compounds. (A) Venn diagram of aroma-active compounds (VIP > 1, OAV > 1, and GC-O). (B) Correlation analysis between sensory attributes and key aroma compounds. (C) Aroma wheel of aroma-active compounds in BX, FZ1, and RX.

By integrating the results from OPLS-DA (VIP > 1), OAV > 1, and GC-O analysis, 17 aroma compounds were identified from the BX, FZ1, and RX samples (Fig. 4A; Table 2). To delve deeper into the correlation between the sensory attributes of each sample and the key aroma compounds, and to identify the key compounds responsible for the formation of the apple aroma, we performed PCA (*r* > 0.8, *p* < 0.05) between the sensory scores from the descriptive sensory analysis and the concentrations of the aforementioned 17 key compounds (Fig. 4B). The results showed that 4 volatile compounds (methylheptenone, *β*-damascenone, (E)-2-decenal and 1-nonanal.) were significantly positively correlated with the fruity attribute; 6 compounds (hexyl acetate, 1-nonanal, 1-nonanol, hexanal, butyl hexanoate, and decyl aldehyde) were significantly positively correlated with the apple-like attribute; and 6 compounds, including decyl aldehyde, hexyl acetate, and hexanal, were positively correlated with the green attribute. It is noteworthy that the sensory results identified the aroma profile of the FZ1 sample as being dominated by an apple-like note, accompanied by a fresh green note, which aligns with typical green apple aroma characteristics. Therefore, we focused our investigation on the volatile components significantly associated with both the apple-like and green attributes. An aroma wheels was constructed for FZ1, BX, and RX (Fig. 4C; Table S6) to provide a systematic and intuitive characterization of their overall aroma profiles.

**Table 2:** GC-O of key aroma-active compounds in BX, FZ1, and RX.

No.	CAS#	Compounds	Odor description	Aroma intensity		
				BX	FZ1	RX
1	109-21-7	Butyl butyrate	Fruity, banana	3.20	1.50	1.00
2	110-93-0	Methylheptenone	Citrus, green	2.30	1.60	3.20
3	112-06-1	Heptyl acetat	Fresh, fruity	2.00	–	–
4	112-31-2	Decyl aldehyde	Sweet, fresh	–	1.00	1.50
5	124-19-6	1-Nonanal	Rose, fresh	1.50	2.00	3.05
6	23726-93-4	*β*-Damascenone	Apple, rose	–	–	2.50
7	142-92-7	Hexyl acetate	Fruity, apple, sweet	–	3.60	4.30
8	143-08-8	1-Nonanol	Rose, citrus	–	1.50	2.00
9	2198-61-0	Isoamyl hexanoate	Banana, apple	2.20	2.69	–
10	6728-26-3	Trans-2-hexenal	Green, grassy	–	4.50	3.80
11	539-90-2	Isobutyl butyrate	Fruity, sweet	2.00	–	–
12	60415-61-4	2-Pentyl Butyrate	fruity, sweet, banana	1.50	2.90	–
13	626-82-4	Butyl hexanoate	Fruity, apple, green	–	2.50	2.00
14	3913-81-3	(E)-2-decenal	Earthy	–	–	1.50
15	659-70-1	Isoamyl valerianate	Fruity, sweet	1.50	2.80	–
16	66-25-1	Hexanal	Fresh, Green, grassy	–	4.80	4.00
17	106-27-4	Isoamyl butyrate	Fruity	3.68	2.00	0.00

Note: Odor thresholds used for OAV calculation were obtained from Van Gemert (the second, extended edition of Compilations of Odor Threshold Values in Air, Water and Other Media). Odor descriptions were sourced from The Good Scents Company (http://www.thegoodscentscompany.com) and Perflavory (http://perflavory.com/), with cross-verification against the Odor Database (http://www.odour.org.uk/) and the Food Flavor Laboratory database (http://foodflavorlab.cn/).

Finally, we identified three key aroma compounds: hexyl acetate, trans-2-hexenal, and hexanal. These three compounds were detected only in the RX and FZ1 samples and were absent in BX. Given their strong association with apple aroma and their specific presence/absence pattern across samples, they were preliminarily identified as the core contributors to the green aroma in FZ1. This hypothesis requires further verification through subsequent studies such as addition experiments.

### 3.6 Molecular docking analysis of key aroma compounds with olfactory receptors

Molecular docking has been proven to be an effective research tool for elucidating the action mechanisms of key flavor compounds and the formation mechanisms of flavor (Zhang et al., 2025). Accordingly, this study systematically evaluated the interaction patterns of three key volatile compounds—hexyl acetate, trans-2-hexenal, and hexanal—as ligands with 564 human olfactory receptors (Table S7). The receptor sequences were retrieved from the UniProt database (https://www.uniprot.org), and their three-dimensional structures were built by homology modeling using templates with known experimental structures, following the same strategy adopted in recent odorant–receptor interaction studies (Wu et al., 2025; Zhang et al., 2025).

**Fig. 5:**
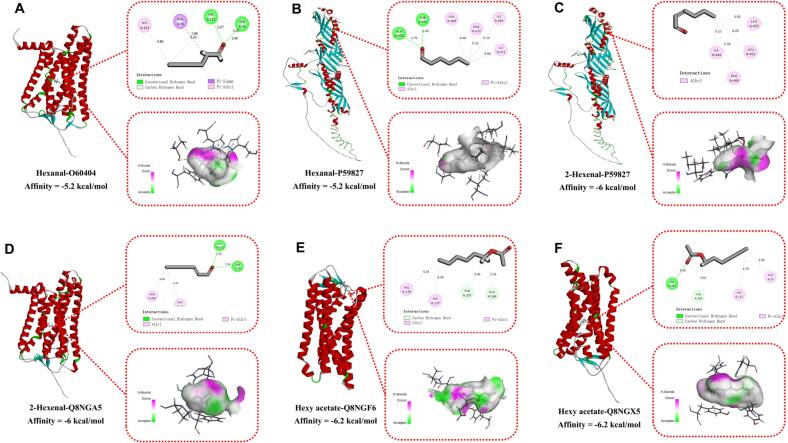
Molecular docking analysis of key aroma compounds with olfactory receptors. Binding modes and interaction mechanisms for (A) Hexanal-O60404, (B) Hexanal-P59827, (C) trans-2-hexenal-P59827, (D) trans-2-hexenal-Q8NGA5, (E) Hexyl acetate-Q8NGF6, and (F) Hexyl acetate-Q8NGX5 complexes.

The results showed a variation in binding energies between the receptors and the volatile compounds (Fig. 5; Table S7), with binding free energies ranging from −6.2 to −0.3 kcal/mol. In the docking score ranking, hexyl acetate had the lowest binding energy with receptors Q8NGF6 and Q8NGX5 (−6.2 kcal/mol), trans-2-hexenal had the lowest binding energy with P59827 and Q8NGA5 (−6.0 kcal/mol), and hexanal had the lowest binding energy with O60404 and P59827 (−5.2 kcal/mol). These top-ranked receptors represent computationally predicted targets for the apple-like and green aroma; although direct experimental binding data for these specific pairs are not yet available, the observed binding modes, dominated by hydrophobic interactions and hydrogen bonds, are fully consistent with the structural principles of odorant recognition recently revealed for a human odorant receptor by cryo-EM (Billesbølle et al., 2023).

The differences in binding affinity were closely associated with the molecular structures of the compounds, particularly influenced by functional group distribution and hydrophobic properties (Kelly & Mancera, 2005). The binding mechanism of hexanal with the O60404 receptor involved a close collaboration between hydrogen bonds and hydrophobic interactions (Fig. 5A). Specifically, its aldehyde oxygen atom formed hydrogen bonds with SER70 (2.26 Å) and HIS112 (2.07 Å), while the terminal part of its alkyl chain approached HIS112 (4.43 Å), PHE74 (2.28 Å), and HIS253 (4.80 Å), forming Pi-Alkyl and Pi-sigma interactions. In its interaction with P59827 (Fig. 5B), its aldehyde group formed stable hydrogen bonds with THR525 (2.45 Å) and ALA502 (2.76 Å), while its carbon terminus interacted with multiple residues via hydrophobic contacts. These multiple interactions collectively built a stable binding microenvironment, enhancing the positioning accuracy and conformational stability of the ligand within the receptor pocket. The synergistic mode of hydrogen bonding and hydrophobic interactions may prolong the residence time of the small molecule in the olfactory protein, thereby enhancing the persistence and recognition efficiency of the perceptual signal.

The binding mechanism of trans-2-hexenal also demonstrated a synergistic effect between hydrogen bonding and hydrophobic interactions (Fig. 5C-D). Its aldehyde carbonyl group engaged in hydrophobic interactions with residues SER70 (2.36 Å) and HIS112 (2.20 Å), thereby enhancing the stability of the binding conformation. Simultaneously, the alkene chain of trans-2-hexenal formed hydrophobic interactions around PRO285 (4.00 Å) and PHE74 (4.41 Å), further stabilizing the binding conformation. In its binding with P59827, trans-2-hexenal primarily contacted non-polar residues such as LEU452 (4.25 Å), LEU455 (3.92 Å), ILE444 (5.33 Å), and PHE449 (5.11 Å) through hydrophobic interactions, forming a stable hydrophobic encapsulation effect. This multi-site synergistic binding mechanism allows trans-2-hexenal to maintain a stable conformation within the protein receptor, thus playing an important role in olfactory recognition and aroma signal transduction.

The binding sites of hexyl acetate primarily involved hydrophobic amino acid residues, including TYR257 (2.45 Å), TYR73 (3.90 Å), Glu166 (2.35 Å), etc. (Fig. 5E-F). The ester group and non-polar alkyl chain in its molecular structure help form strong and stable interactions with these hydrophobic residues. Furthermore, except for the complex formed between trans-2-hexenal and P59827, hydrogen bond formation was observed in all other complexes. This synergistic effect of hydrophobic interactions and hydrogen bonding forms the structural basis for their high binding affinity.

In summary, these three key aroma compounds form stable interactions with specific olfactory receptors through various intermolecular forces. The synergistic effects of hydrophobic interactions and hydrogen bonding explain the stable binding mechanism of these aroma compounds to their olfactory targets (Zhang et al., 2025), providing key insights into the molecular mechanism of aroma perception. These findings not only deepen our understanding of how apple aroma attributes are perceived at the receptor level but also highlight the core importance of molecular interactions in shaping sensory attributes.

### 3.7 Molecular dynamics simulation reveals stable binding of key aroma compounds to olfactory receptors

**Fig. 6:**
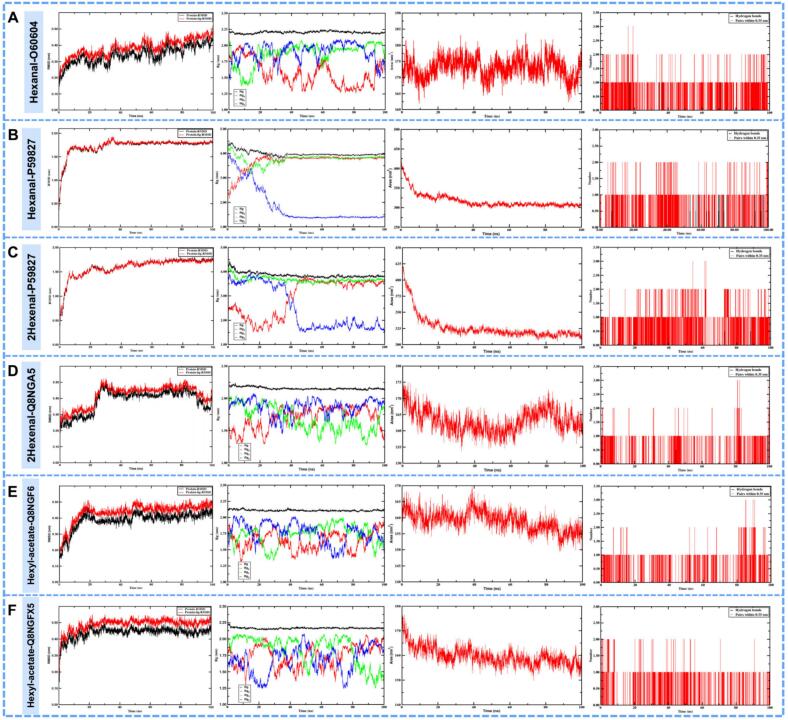
Molecular dynamics simulation of aroma compound-olfactory receptor complexes. Stability analysis of (A) Hexanal-O60404, (B) Hexanal-P59827, (C) Trans-2-hexenal-P59827, (D) Trans-2-hexenal-Q8NGA5, (E) Hexyl acetate-Q8NGF6, and (F) Hexyl acetate-Q8NGX5 complexes through 100 ns simulations, showing RMSD, Rg, and SASA profiles.

While the binding free energies obtained from molecular docking indicate strong initial affinity and favorable binding poses, static docking scores alone are insufficient to confirm dynamic structural stability. Therefore, to elucidate the dynamic binding mechanisms and definitively substantiate the stable binding of key aroma compounds with olfactory receptors, we performed 100 ns molecular dynamics (MD) simulations on the six screened complexes with the highest affinity (hexanal-O60404, hexanal-P59827, trans-2-hexenal —P59827, trans-2-hexenal-Q8NGA5, hexyl-acetate-Q8NGF6, and hexyl-acetate-Q8NGX5) to evaluate their conformational stability and binding mechanisms (Fig. 6A–F).

The root mean square deviation (RMSD) is a key indicator for assessing the stability of protein-ligand complexes; a stable RMSD curve indicates a more stable complex. RMSD analysis of the six complexes (Fig. 6) showed that all systems reached equilibrium after 50 ns. Among them, hexanal-O60404 (0.38 ± 0.05 Å), trans-2-hexenal-Q8NGA5 (0.42 ± 0.07 Å), hexyl-acetate-Q8NGF6 (0.44 ± 0.06 Å), and hexyl-acetate-Q8NGX5 (0.49 ± 0.04 Å) had low RMSD values, indicating minimal protein backbone fluctuations and exceptionally stable complex structures. In comparison, hexanal-P59827 (1.73 ± 0.18 Å) and trans-2-hexenal-P59827 (1.59 ± 0.20 Å) had relatively higher RMSD values, but their curves also stabilized after equilibrium, suggesting stable binding nonetheless.

The radius of gyration (Rg) is a commonly used parameter to assess the compactness of protein structures, reflecting protein stability; lower Rg values indicate a more stable structure (Zhang et al., 2025). The Rg values for the six complexes were 2.20 ± 0.02 nm, 4.00 ± 0.11 nm, 3.86 ± 0.11 nm, 2.15 ± 0.02 nm, 2.10 ± 0.01 nm, and 2.16 ± 0.12 nm, respectively (Fig. 6A–F). The results indicate that the complexes bound to P59827 had looser structures, whereas the complexes bound to O60404, Q8NGA5, Q8NGF6, and Q8NGX5 exhibited more compact, globular structures.

The solvent accessible surface area (SASA) is another important parameter for evaluating protein folding and stability. The SASA results for the six complexes were highly consistent with the Rg trend (Fig. 6A–F). The SASA values for the six complexes were 172.94 ± 3.13 nm^2^, 317.52 ± 19.28 nm^2^, 326.25 ± 19.00 nm^2^, 163.10 ± 3.50 nm^2^, 159.05 ± 3.06 nm^2^, and 159.72 ± 3.69 nm^2^, respectively. The first two complexes had larger SASA values, while the latter four had smaller and stable SASA values, indicating that no significant structural changes, internal system exposure, or folding alterations occurred during the simulation.

The stability of intermolecular interactions relies on a persistent hydrogen bond network. Analysis of the number of hydrogen bonds (Fig. 6A–F) showed that all complexes maintained a stable and considerable number of hydrogen bonds throughout the simulation, providing dynamic support for the key hydrogen bond interactions observed in the molecular docking. This persistent hydrogen-bond network is a decisive factor for maintaining the precise positioning of the ligand in the receptor binding pocket and the high stability of the complexes.

In summary, while molecular docking provided initial predictions of binding affinity, the molecular dynamics simulations confirmed from a dynamic perspective that the key apple-like aroma compounds can form stable complexes with their corresponding human olfactory receptors. Although the geometric characteristics of different receptor binding pockets led to variations in structural compactness, all systems exhibited excellent overall stability and sustained intermolecular interactions, providing robust dynamic evidence for their potential biological role in olfactory perception. This coupled docking and molecular dynamics approach is a well-established standard for substantiating stable binding interactions in flavor chemistry (Billesbølle et al., 2023).

### 3.8 Aroma addition experiment

**Fig. 7:**
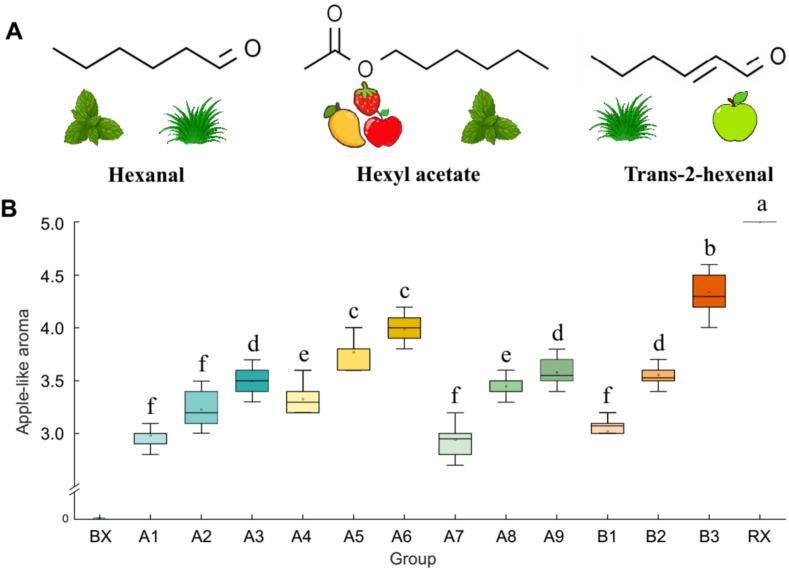
Sensory validation of key aroma compounds through addition experiments. (A) Molecular structures and odor characteristics of hexanal, trans-2-hexenal, and hexyl acetate. (B) Sensory evaluation scores of apple-like aroma intensity in banana matrix supplemented with individual compounds and their combination at low (L), medium (M), and high (H) concentrations.

Based on the above experiments, we identified hexyl acetate, trans-2-hexenal, and hexanal as three key compounds constituting the apple-like aroma (Fig. 7A). To verify their sensory contribution, we designed three addition levels based on the natural concentration differences of these compounds in the BX, FZ1, and RX samples: L (half concentration), M (1× concentration), and H (2× concentration).

The results of single-compound addition experiments showed that all three compounds could enhance the intensity of the apple-like aroma (Fig. 7B), and the sensory scores were concentration-dependent. The effect of trans-2-hexenal was the most significant (L: 3.33; M: 3.77; H: 3.99), while the enhancement effects of hexanal (L: 2.94; M: 3.45; H: 3.61) and hexyl acetate (L: 2.98; M: 3.23; H: 3.50) were similar. Notably, at the low concentration (L), the effects of hexyl acetate and hexanal on the apple like aroma intensity were not obvious, whereas at the high concentration (H), all three compounds significantly enhanced the green apple like aroma characteristics. When the three compounds were added together, a significant additive effect was observed: even at the half concentration (L: 3.03), the aroma was effectively enhanced, and the magnitude of enhancement at the high concentration (H: 4.34) surpassed that of any single compound.

The addition experiments proved that all three compounds can enhance the apple-like aroma in banana. Among these, hexanal and trans-2-hexenal are both aldehydes, products of the LOX pathway, primarily contributing green, grassy, leafy, and fresh aromas. However, Hexanal tends towards a strong green note, while trans-2-hexenal is more subtle, presenting as leafy and fruity base notes. In this study, the enhancing effect of trans-2-hexenal was more prominent, potentially due to additive effects when interacting with other flavor substances in the matrix. Hexyl acetate, as an ester compound, directly provides a pleasant sweet-fruity aroma with a fresh green or leafy undertone, and is an important source of the fruity note in many fruits (e.g., apples, pears, strawberries) and fruit-flavored foods (Landy et al., 1996). Although its effect when added alone was relatively weak, it played a key role when combined with the two aldehydes: the effect was best when all three compounds were added at high concentrations. This combination not only enhanced the richness and fruitiness of the aroma but also effectively balanced the somewhat pungent green note imparted by hexanal, resulting in a more balanced and pleasant overall aroma profile closer to that of green apple. While these addition models confirm their strong capacity to impart this aroma, future omission experiments will be necessary to definitively establish their absolute indispensability to the natural FZ1 aroma.

Previous studies have suggested that hexanal and trans-2-hexenal are important volatile aroma components in apple fruits. These compounds are mainly generated through the lipoxygenase (LOX) pathway and contribute significantly to the green or leafy aroma characteristics of apples (Risticevic et al., 2020). Analysis of 35 apple cultivars revealed that hexanal is one of the key characteristic aroma-active compounds in apples, and its concentration and odor threshold significantly influence the overall aroma perception of apples (Wu & Bi, 2022). The present study extends this conclusion to the banana flavor system, indicating that these aldehyde compounds can also enhance the perception of green apple-like aroma in banana. During apple ripening, the change in (*E*)- trans-2-hexenal content is closely related to the aroma development of the fruit, especially in the early stages of ripening, playing an important role in forming the fresh, green aroma profile of apples (Contreras & Beaudry, 2013). In this study, it also significantly enhanced the perception of the apple-like aroma in banana. Although hexyl acetate is widely recognized as a key aroma compound in apples (Moya-Leon et al., 2004), this study found its individual effect in the banana system was weak, suggesting that its aroma contribution is matrix-dependent. This finding provides a new perspective for understanding flavor formation mechanisms in complex food matrices.

## 4 Conclusion

In conclusion, this study examined the chemical and sensory basis of the apple-like aroma in the banana cultivar ‘Fenza 1’ (FZ1). Untargeted GC–MS, GC-O, and multivariate analysis were used to identify odor-active metabolites associated with this aroma. Spike-in experiments with hexyl acetate, trans-2-hexenal, and hexanal supported their contribution to the apple-like perception and indicated an additive effect when these compounds were combined, enabling partial reconstitution of the aroma in a banana matrix. The perception of this aroma was further explained at the molecular level: molecular docking and molecular dynamics simulations confirmed that these compounds form stable complexes with specific human olfactory receptors, primarily mediated hydrophobic interactions and hydrogen bonds.

However, several methodological boundaries remain to be addressed in future research. To capture the dynamic temporal release of volatiles during human mastication, which static HS-SPME cannot assess, in vivo flavor release techniques like PTR-TOF-MS should be deployed. In terms of sensory methodology, rigorous omission experiments will be required to verify the absolute indispensability of these odorants. Lastly, while our in silico models provide strong predictive value, they cannot fully replicate the human nasal mucosa. Therefore, future studies must prioritize in vitro cellular assays to biologically validate these receptor interactions, concurrent with investigations into the upstream genetic regulatory networks driving the biosynthesis of these target compounds. Together, these results provide a basis for understanding aroma formation in FZ1 and may be useful for future efforts to improve banana flavor quality and develop natural banana flavorings.

## CRediT authorship contribution statement

**Wen Zeng:** Writing – original draft, Investigation, Funding acquisition, Formal analysis. **Zhiwen Zeng:** Methodology, Investigation, Formal analysis. **Tao Wang:** Investigation, Formal analysis. **Yacong Hou:** Investigation, Formal analysis. **Youfeng Jiang:** Investigation. **Dafeng Dong:** Formal analysis. **Tianxiang Li:** Investigation. **Lufeng Fu:** Investigation. **Ying Liu:** Supervision, Investigation. **Xuesong Cao:** Supervision, Methodology. **Zhuo Chen:** Supervision, Investigation, Formal analysis. **Peitao Lü:** Writing – review & editing, Investigation, Funding acquisition, Formal analysis.

## Funding

This work was supported by the Project of State Key Laboratory of Tropical Crop Breeding (SKLTCBBSH202505 and NKLTCBCXTD26), 10.13039/501100004761Natural Science Foundation of Hainan Province (326JCQN0983), the 10.13039/501100010038Earmarked Fund for Agriculture Research System of China (CARS-31).

## Declaration of competing interest

The authors declare that they have no known competing financial interests or personal relationships that could have appeared to influence the work reported in this paper.

## Data Availability

Data will be made available on request.
