# Supplementary figures and images for: Chemical and sensory characterization of the apple-like aroma in ‘Fenza 1’ banana unveiled by sensory-directed analysis and molecular modeling
Source: Food Chem X. 2026 May 28;36:104047. doi: 10.1016/j.fochx.2026.104047 (PMC13233587; doi:10.1016/j.fochx.2026.104047)

## Supplementary Figure 1

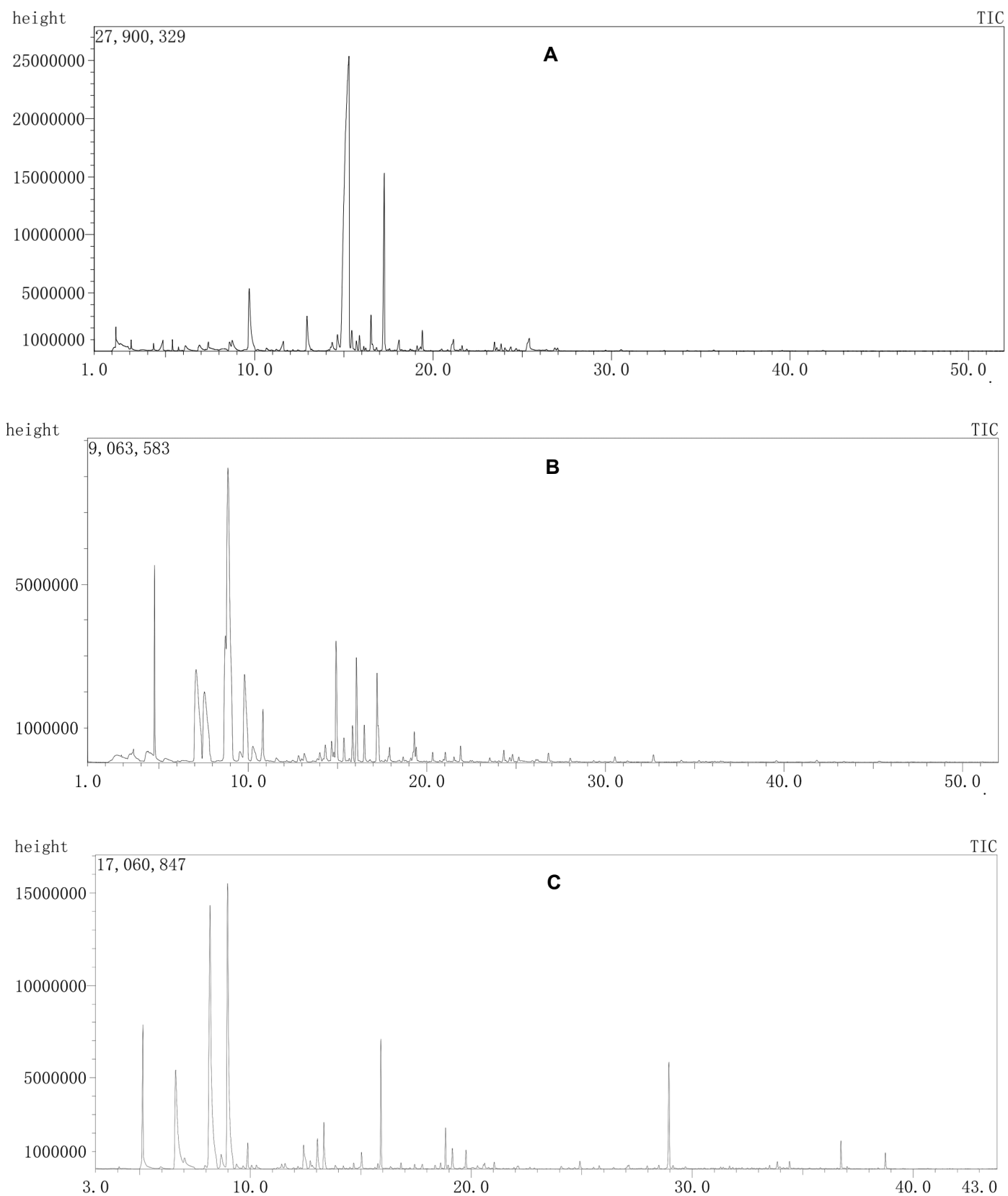

**Fig. S1.** Total ion chromatogram (TIC) of BX (A), FZ1 (B), and RX (C)

Supplement: Supplementary material 1 [file mmc1.pdf]
